# Cloning and Functional Analysis of *Pax6* from the Hydrothermal Vent Tubeworm *Ridgeia piscesae*

**DOI:** 10.1371/journal.pone.0168579

**Published:** 2016-12-22

**Authors:** Huifang Yuan, Wei Wang, Bin Hu, Changkun Pan, Mingliang Chen, Linlin Ke, Lirong Yang, Jianming Chen

**Affiliations:** 1 Key Laboratory of Marine Biogenetic Resources, Third Institute of Oceanography, State Oceanic Administration, Xiamen, Fujian Province, China; 2 School of Marine Sciences, Ningbo University, Ningbo, Zhejiang, China; Laboratoire Arago, FRANCE

## Abstract

The paired box 6 (*Pax6*) gene encodes a transcription factor essential for eye development in a wide range of animal lineages. Here we describe the cloning and characterization of *Pax6* gene from the blind hydrothermal vent tubeworm *Ridgeia piscesae* (*RpPax6*). The deduced RpPax6 protein shares extensive sequence identity with Pax6 proteins from other species and contains both the paired domain and a complete homeodomain. Phylogenetic analysis indicates that it clusters with the corresponding sequence from the closely related species *Platynereis dumerilii* (*P*. *dumerilii*) of Annelida. Luciferase reporter assay indicate that RpPax6 protein suppresses the transcription of *sine oculis* (*so*) in *D*. *melanogaster*, *interfering* with the C-terminal of RpPax6. Taking advantage of *Drosophila* model, we show that RpPax6 expression is not able to rescue small eye phenotype of ey^2^ mutant, only to cause a more severe headless phenotype. In addition, *RpPax6* expression induced apoptosis and inhibition of apoptosis can partially rescue *RpPax6*-induced headless phenotype. We provide evidence *RpPax6* plays at least two roles: it blocks the expression of later-acting transcription factors in the eye development cascade, and it promotes cell apoptosis. Our results indicate alternation of the *Pax6* function may be one of the possible causes that lead the eye absence in vestimentiferan tubeworms.

## Introduction

Members of the paired box *(Pax*) gene family encode transcription factors that are characterized by a DNA-binding paired domain of 128 amino acids located at the amino terminal end [1]. Additionally, some Pax proteins contain a partial or complete paired-type homeodomain and/or an octapeptide motif. Members of the *Pax* gene family are involved in the regulation of wide range of developmental processes, including segmentation and organogenesis [2, 3]. *Pax6* is one of the well-characterized *Pax* genes which plays critical role in eye development in diverse animal lineages. It contains both a paired and a complete homeodomain. The genomic organization, domain sequences, and function are highly conserved [4, 5].

Mutations in the *Pax6* gene cause aniridia (a panocular disorder primarily characterized by complete or partial absence of iris tissue) in human and small eye (*sey*) phenotype (microphthalmia and small body size) in mouse [6, 7]. Mutations in the *Drosophila Pax6* homolog eyeless (*ey*) cause partial to complete loss of the compound eye as well as surrounding sensory bristles. Moreover, misexpression of *Pax6* genes from many divergent species including *Drosophila* [8], ribbon worm [9], squid [10] and ascidian [11] induce ectopic eyes in *Drosophila*. In frogs, *Pax6* misexpression can also induce ectopic eyes [12]. The conservation of regulatory cascade required for eye morphogenesis is further supported by the fact that *eya*-Eya, *so*-Six, and atonal-*Ath5* gene families all act immediately downstream of *Pax6* in both fruit fly and mouse eye development [13–16], indicating the *Pax6* dependent eye developmental pathway can be traced at least in the last common ancestor of protostome and deuterostome (urbilateria) and has been adapted to the control of development of different visual systems found in both clades.

The comparative analyses of bilaterian eyes have revealed that the simplest morphology comprises one photorceptor cell and one pigment cell [17]. In Annelida, two distinct types of pigmented cerebral eyes were characterized: larval and adult eyes. Adult annelid eyes are multicellular, whereas larval annelid eyes are comparatively simple, usually only comprising of two cells: one rhabdomeric photoreceptor cell and one pigment cell. Larval-type eyes match the prototypical two-celled eye and are present in larvae of species of many protostomian lineages [18, 19, 20] as well as deuterostomian lineages including hemichordates [21] and cephalochordates [22]. The larval eyes are usually replaced by the eyes of the adults during later development [23–26]. The adult multicellular eyes show a very characteristic structure with photoreceptor cell processes traversing the pigment cell layer. This type of eyes is found widespread in various protostomian lineages including polychaetes [23, 27], molluscs [28], sipunculans [29] and onychophorans [30]. Both larval and adult eyes are molecularly characterized in the marine annelid *Platynereis dumerilii*. *Pax6* was found to be expressed in the larval but not adult eyes of *P*. *dumerilii* [23].

Based the resemblance to the fossilized tubes, modern vestimentifera tubes old as 430 million years form a derived clade of vestimentifera within the annelid radiation.” [31]. Vestimentiferans are important members of deep-sea chemosynthetic communities, which include hydrothermal vents, cold seeps, whale falls and reduced sediments. Due to their deep-sea habitats the first member of vestimentifera *Siboglinum weberi* was not reported until early in the 20th century [32]. More than 100 species of vestimentiferans, have been described. Like many deep-sea organisms as well as cave-dwelling animals, the vestimentiferans tubeworms lack eyes. They rely on microbial endosymbionts for their energetic needs. Vestimentiferan tubeworms have been extensively studied though research has focused primarily on phylogeny and bacterial symbionts [33–35]. Far fewer studies have explored the molecular machinery used in the regulation of development and innate immune system.

To further our understanding of the biomolecular mechanisms underlying the eye absence in vestimentifera lineage, we isolated *Pax6* gene of vestimentiferan *Ridgeia piscesae*. *R*. *piscesae is* the foundation specie in many Juan de Fuca Ridge hydrothermal vent communities [36, 37]. In order to investigate the function of the *RpPax6*, the model organism *D*. *melanogaster* was applied for genetic analysis. *D*. *melanogaster* is accessible for a broad range of genetic and molecular techniques and has been widely used for the elucidating the function of *Pax6* from various species. RpPax6 protein is found to be able to suppresses the transcription of *sine oculis* (*so*) which can interfere with the activation of eye development network. In addition *RpPax6* is associated with the elevated level of apoptosis. Our results suggest that functional alternation of *RpPax6* may be involved in the eye absence in vestimentiferan tubeworms.

## Materials and Methods

### cDNA cloning of *R*. *piscesae Pax6*

The cDNA library of *R*. *piscesae* was previously constructed [38]. Sequences of cDNA clones were analyzed and a clone bearing putatively entire ORF of *Pax6* was identified. To obtain the full length cDNAs of *Pax6* gene, the 3’ and 5’ ends were obtained by rapid amplification of cDNA ends (RACE) approaches using 3’ -Full RACE Core Set with PrimeScript™ RTase and 5’ -Full RACE Kit with TAP (TaKaRa, Japan) following the manufacturer’s instructions. The PCR products were ligated into pMD-19T vector (TaKaRa, Japan) and transformed into the competent *E*. *coli* TOP10 cells. Positive clones with the expected-size inserts were determined by colony PCR and DNA sequencing.

### Phylogenetic analysis

Accession numbers for sequences included in the analyses are: *Doryteuthis opalescens Pax6* (*DoPax6*): AAB40616; *Euprymna scolopes Pax6* (*EsPax6*): AAM74161; *Crassostrea gigas Pax6* (*CgPax6*): XP_011433289; *Idiosepius paradoxus Pax6* (*IpPax6*): BAM74253; *Ambystoma mexicanum Pax6* (AmPax6): AAD50903; *Anolis carolinensis Pax6* (*AcPax6*): XP_008104750; *Cavia porcellus Pax6* (*CpPax6*): XP_003464531; *Aotus nancymaae Pax6* (*AnPax6*): XP_012307699; *Xenopus laevis Pax6* (*XlPax6*): AF154555; *Homo sapiens Pax6* (*HsPax6*): NP_000271; *Platynereis dumerilii Pax6* (*PdPax6*): CAJ40659; *Terebratalia transversa Pax6* (*TtPax6*): ADZ24784; *Lottia gigantea Pax6* (*LgPax6*): XP_009066032; *Cupiennius salei Pax6* (*CsPax6*): CEH19758; *Saccoglossus kowalevskii Pax6* (*SkPax6*): NP_001158383; *Limulus polyphemus Pax6* (*LpPax6*): XP_013778820; *Drosophila melanogaster eyeless* (*DmEy*): AAF59318; *Mus musculus Pax2* (*MmPax2*): CAA39302.1. The amino acid sequences of Pax proteins were aligned and a phylogenetic tree was generated using the Mega 3 [39]. The neighbor joining method with 1000 bootstrap replications was used.

### Fly stocks

The following fly stocks were used:

y w;+/+;ey-GAL4/TM6B,Tb
y w;+/+;GMR-GAL4
y w;+/+;dpp-GAL4/TM6B,Tb
y w;+/+;da-GAL4
y w;+/+;da-GAL4,tub-GAL80^ts^
y w;+/+;UAS-RpPax6
y w;+/+;UAS-ey
y w;UAS-P35/cyo,y+;FRT-82B
y w;+/cyo
y w;+/+;+/TM6B,Tb
w t;ey^2^
y w;+/+;UAS-mPax6/Tm3;ey^2^

### Construction of plasmids

We constructed a set of chimeric molecules in which individual segments of ey were deleted and replaced with the corresponding region of RpPax6. The *eyN+RpPax6C* chimera was created by replacing the N terminal of RpPax6 with amino acids 1–471 of ey. The *eyPD+RpPax6HD* chimera was created by replacing the PD of RpPax6 with amino acids 36–164 of ey. Similarly, we generated a set of chimeric molecules in which individual segments of RpPax6 were deleted and replaced with the corresponding region of ey. The *RpPax6PD+eyHD* chimera was created by replacing the PD of ey with amino acids 17–143 of RpPax6. The *RpPax6N+eyC* chimera was generated by replacing the N terminal segment of ey with amino acids 1–296 of RpPax6.

The firefly luciferase reporter plasmid (pGL3-so and pGL3-eya) were constructed by inserting the 428 bp of *so10* and a 398 bp of *so5* fragments [13] and -499 to +100 fragment eya gene promoter region PCR amplified from the *Drosophila* genome in pGL3-basic vector (Promega, America) at *Kpn* I and *Xho* I sites. The plasmid pUAST-RpPax6 was generated by subcloning the *RpPax6* cDNA into the *Drosophila* transformation vector pUAST using *EcoR* I and *Xho* I. The transgenes encoding wild-type and mutated RpPax6 and ey proteins were constructed as follows: pCMV-myc-RpPax6, pCMV-myc-ey, pCMV-myc-RpPax6N+eyC, pCMV-myc-eyN+RpPax6C, pCMV-myc-eyPD+RpPax6HD, pCMV-myc-RpPax6PD+eyHD were prepared by subcloning the inserts from the corresponding pEASY-T5 vectors into vector pCMV-myc plasmid (Clontech, Japan) using *EcoR* I and *Xho* I. Several transgenic lines of each construct were obtained by P-element-mediated germline transformation according to standard procedures. All of the constructs were confirmed by DNA sequencing.

### Temperature-shift experiments

Conditional expression of RpPax6 was carried out by using *y w;+/+;UAS-RpPax6/da-GAL4*, *tub-GAL80*^*ts*^ line. GAL80^ts^ is a temperature sensitive form of the GAL80 repressor. At 25°C degrees, the GAL80^ts^ represses the activity of GAL4; however, when the temperature is shifted to 29°C degrees, the GAL80^ts^ is inactivated, allowing for the GAL4 transcription factor to bind to the UAS binding sites and express the gene of interest [40]. Larvae were raised at 18°C (GAL80^ts^ permissive temperature) for 120 h then shift to 29°C (GAL80^ts^ restrictive temperature) for 24 h. Twenty third instar larva were collected. Larger discs (including the wing and eye-antenna discs) were dissected and incubated for 5 minutes in 5 g/mL acridine orange in phosphate-buffered saline (PBS) [41]. The organs were then placed in fresh PBS and analyzed immediately for nuclear staining on a Leica M165FC fluorescence stereomicroscope (Leica, Wetzlar, Germany). Independent triplicate experiments were performed.

### Luciferase assays

Transient transfections of 293T cells were performed in 24-well plates with the transfection reagent Lipofectamine 2000 (Invitrogen, America). For each well, cells were transfected with 100 ng of pSV-β-galactosidase (Promega, America) as transfection efficiency control, 200 ng reporter vector (pGL3-so) and 200 ng expression vector (pCMV-myc-RpPax6, pCMV-myc-ey, pCMV-myc-RpPax6N+eyC, pCMV-myc-eyN+RpPax6C, pCMV-myc-eyPD+RpPax6HD, pCMV-myc-RpPax6PD+eyHD or pCMV-myc). pCMV-myc vector was used as a negative control. At 6 h post-transfection the medium was replaced. Cells were harvested 30 h after transfection, rinsed with PBS, resuspended in reporter cell lysis buffer (Promega, America), and incubated for 10 minutes at room temperature. The lysate was centrifuged at 12,000 × g for 5 min to pellet the cell debris. The supernatants were transferred to a fresh tube. A 10 μL-aliquot of the extract was added to 25 μL of the luciferase assay substrate (Promega, America) and the luminescence of the samples were read immediately on a Glomax™ 20/20 Luminometer (Promega, America). Each transfection was performed in triplicate.

β-Galactosidase activity was used for normalizing the transfection efficiency and protein input. A 10 μL-aliquot of the cell extract was mixed with 290 μL of O-nitrophenyl-β-D-Galactopyranoside (ONPG) solution (880 μg/ml ONPG, 67 mM Na_3_PO_4_, 1 mM MgCl_2_, 45 mM β-mercaptoethanol, pH 7.5) (Sangon Biotech, Shanghai). The absorbance of the mixture was determined at 420 nm after 30 min of incubation at 37°C. Each transfection was performed in triplicate. Results are expressed as means of the ratio between firefly luciferase activity and β-Galactosidase activity whereas control is 100%.

## Results

### Cloning and phylogenetic analysis *RpPax6*

Analyses of the cDNA library from long-skinny *Ridgeia piscesae* identified a putative *Pax6* gene. The cloned full length Pax6 cDNA contained 312 bp of 5’ untranslated region (UTR), 1410 bp of open reading frame (ORF) encoding 470 amino acids, and 650 bp of 3’ UTR. The predicted protein product contains the paired domain and a complete homeodomain, but not an octapeptide, exhibiting the structural features characteristic of the Pax6 protein [5]. Therefore, we designated this gene *RpPax6* (*R*. *piscesae Pax6*). The nucleotide and amino acid sequences have been assigned to the GenBank Accession Number KT380855.

**Fig 1:**
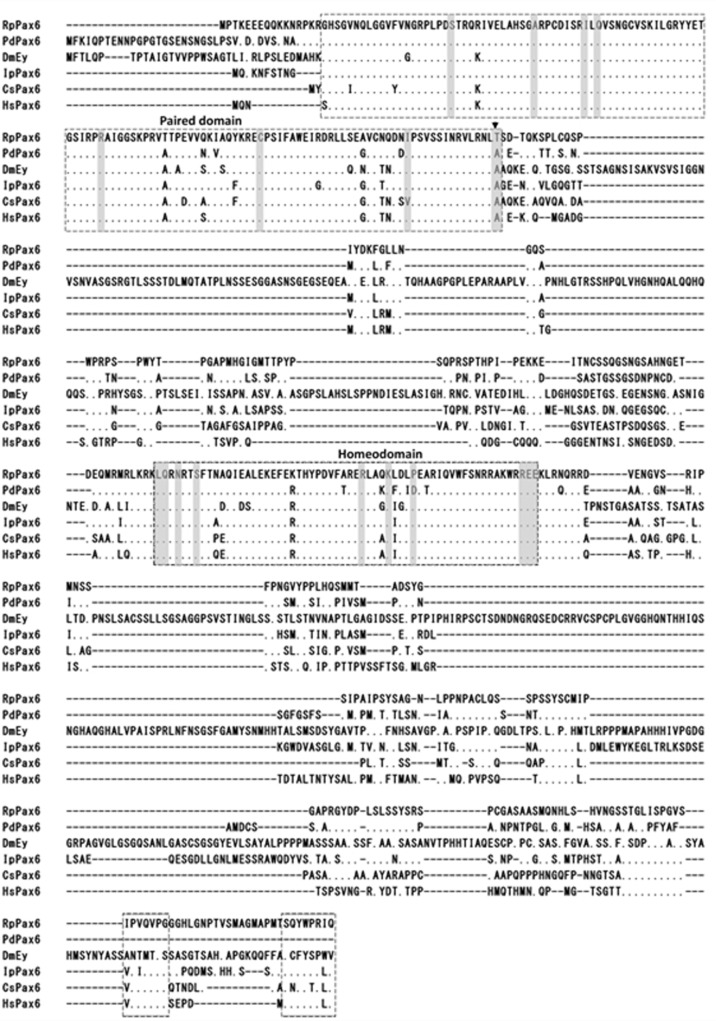
Comparison of amino acid sequences of Pax6 genes. Identical amino acids are indicated by dots. The introduced gaps are indicated by dashes. The paired domain, homeodomain and conserved C-terminal motif are boxed. Pax6-specific amino acids [5] are shaded. Arrowhead indicates a single amino acid change of the Pax6-specific amino acids.

**Fig 2:**
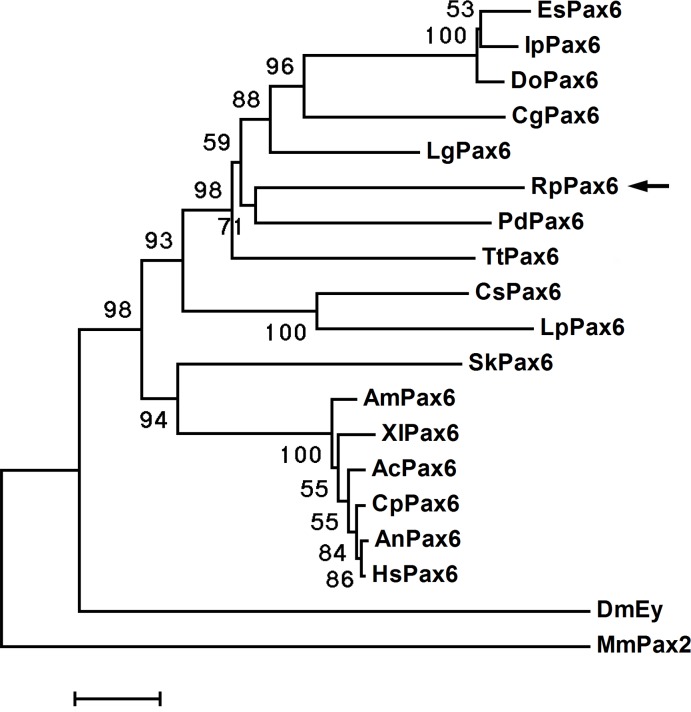
Phylogenetic position of *RpPax6*. A neighbor-joining tree based on a comparison of the deduced amino acid sequences of full-length clones of *Pax6* with mouse Pax2 included as outgroup. Numbers at nodes indicate the levels of bootstrap support based on data for 1,000 replicates; only values greater than 50% are shown. Bar 5% estimated sequenced divergence.

Amino acids sequence alignment of RpPax6 and the pax6 from different species reveal that the paired domains and homeodomains are highly conserved (Fig 1). Positions of Pax6 specific amino acids in the conserved domain [5] are conserved in RpPax6 except one amino acid change. Typical of most paired domains found in Pax6 genes have an asparagine at position 128. However, *RpPax6* encode a threonine at this position. The C-terminal region (C) comprises 152 aa, rich in serine (18%), proline (13%) and asparagine (6%), and contains conserved termination motif. Phylogenetic tree based on full-length Pax6 amino acid sequences was constructed with neighbor joining method (Fig 2). DmEy from *D*. *melanogaster* branched out at the base of the tree other Pax6 proteins were divided into two clades, corresponding protostome and deuterostome respectively. The clade of protostomian lineage comprises two subclades. *Pax6* from lophotrochozoan species form a monophyletic subclade. Among this group the identified *RpPax6* clusters with the corresponding sequence from the closely related species *P*. *dumerilii* of Annelida. The sequences from arthropoda species form the sister group of lophotrochozoan subclade. In the deuterostome clade all vertebrate members of *Pax6* gathered together whereas *Pax6* from hemichordate *S*. *kowalevskii* (*SkPax6*) branch out independently at the basal place.

### *RpPax6* has repressive effect on the promoter activity of *so*

**Fig 3:**
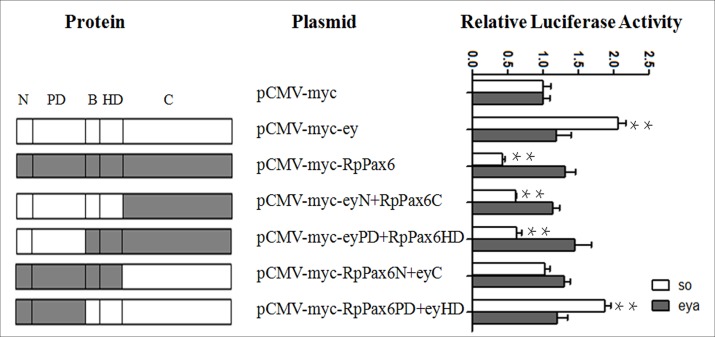
Structural-functional analysis of *ey* and *RpPax6*. Schematic summaries of the original and chimeric constructs are listed in the left column. The right column shows the effect of ey and RpPax6 proteins with or without mutation on promoter activity of pGL3-so and pGL3-eya. The assay was carried out in 293T cells as described in Materials and Methods. Luciferase activities are shown relative to those of pCMV-myc. pSV-β-galactosidase was included in transfection as an internal control of the transfection efficiency. The values are the means from three independent experiments ±SE. **P < 0.01.

RpPax6 shares 91.4% and 88.3% identity at the paired domain and homeodomain with the ortholog of *Drosophila ey* at the amino acid level. *Drosophila* ey protein has been known as a critical regulator of early retinal development and *sine oculis* (*so*) is the direct target of *ey* [42]. In addition the eye-specific enhancer of eya is known to be induced in response to ectopic expression of ey [43]. To test if *RpPax6* is involved in the transcriptional regulation of *ey* downstream target, we examined the effect of RpPax6 and ey proteins with or without mutation on the transcriptional activity of *so* and *eya*. Reporter vectors (pGL3-so and pGL3-eya) were constructed by cloning *so* and *eya* promoters upstream of a luciferase reporter. 293T cells were transfected with luciferase reporter vector and an expression vector bearing chimeric ey and RpPax6 constructs. As demonstrated in Fig 3, neither ey, RpPax6 or chimeric fusion proteins has significant effect on the promoter activity of pGL3-eya, suggesting that ey acts through other factors to regulate eya. Meanwhile, cotransfection of the expression vector pCMV-myc-ey and the pCMV-myc-RpPax6PD+eyHD with the report construct pGL3-so resulted in two-fold increase in the luciferase activity as compared with the level observed when cotransfecting with control pCMV-myc plasmid, indicating that *so* promoter was activated by *ey* and *RpPax6PD+eyHD*. In contrast the expression of *RpPax6*, *eyN+RpPax6C* and *eyPD+RpPax6HD* resulted in significant suppression of luciferase activity, indicating *RpPax6* and *eyN+RpPax6C eyPD+RpPax6HD* have repressive effect on the promoter activity of *so*. Compared with the full-length *RpPax6*, *RpPax6* with paired domain swapped with the corresponding portion of *ey* (*eyN+RpPax6C* and *eyPD+RpPax6HD*) had a much lesser effect on *so* promoter. In the case of *RpPax6* with C region replaced by the counterpart of *ey*, the repressive effect of *RpPax6* was not detected. These results indicate that RpPax6 protein suppresses the transcription of *so* and the C region of *RpPax6* plays crucial role in the repressive activity.

### Ectopic expression of *RpPax6* in *D*. *melanogaster*

**Fig 4:**
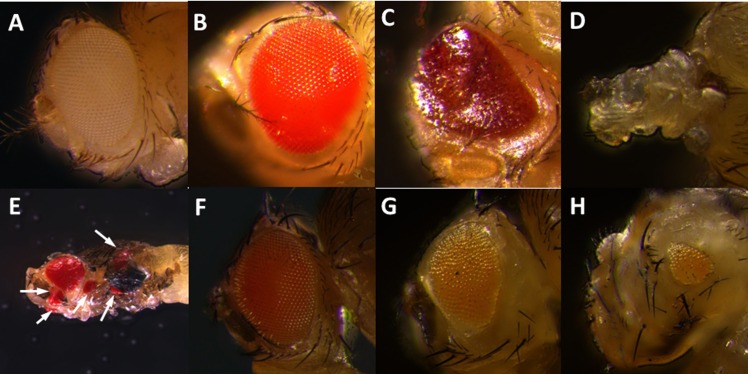
Eye phenotypes caused by misexpression of *RpPax6* and *ey* genes. (A, B) head of *y w* and *UAS-RpPax6* pharate. (C) Rough eye in a *UAS-RpPax6/GMR-GAL4* pharate. (D) *UAS-RpPax6/ey-GAL4* pharate with most head structures and both eyes absent. (E) Ectopic eyes in *UAS-ey/dpp-GAL4 fly* (arrows). (F) Head of *UAS-ey* pharate. (G, H) Right and left eye of *UAS-ey/ey-GAL4* pharate. The left eye was significantly reduced in size and the right eye was slightly reduced.

**Fig 5:**
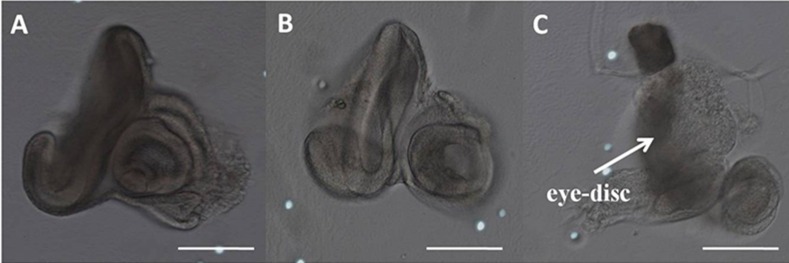
Third instar eye-antenna discs. (A): *y w* (B): *UAS-RpPax6* (C): *UAS-RpPax6/ey-GAL4* flies. The boxed areas show growth defects in the eye disc of UAS-RpPax6/ey-GAL4 larvae. Scale bars = 100 μm.

The GAL4/UAS binary system was used to drive the expression of *RpPax6* in various *Drosophila* tissues. Four GAL4 drivers including *eyeless-GAL4 (ey-GAL4)*, *GMR-GAL4*, *decapentaplegic*-*GAL4* (*dpp-GAL4*), and *daughterless-GAL4* (*da-GAL4*) were employed (Table 1). The *ey-GAL4* driver line expresses GAL4 ubiquitously in the eye-antenna discs throughout early larval development when all the cells are proliferating. During the third instar larval stage, the expression is restricted to the cycling cells anterior to the morphogenetic furrow [8, 44].*GMR-GAL4* expresses behind the morphogenetic furrow in the eye disc. *dpp-GAL4* directs GAL4 expression in all of the imaginal discs throughout development, and in only a limited portion of each disc [45]. *da-GAL4* is used to express GAL4 ubiquitously. We observed early lethality in flies expressing *RpPax6* under the control of all four GAL4 driver lines. Global expression of the *RpPax6* using *da-GAL4* leads to embryonic lethality. Expression of *RpPax6* using *dpp-GAL4* caused lethality during larval and pupal stages. Dissection of the pupae showed the animals could not start metamorphosis of the eyes, wings and legs. Expression of *RpPax6* using *ey-GAL4* exhibits pupal lethality. Examination of these dead pharate adults in the pupal case revealed that most head structures and both eyes were missing (Fig 4D). Dissection of the third instar larvae showed abnormal morphology of eye-antennal discs (Fig 5C). Expression of *RpPax6* using *GMR-GAL4* results pupal lethality with eclosion rate of 7.9%. The pharates and adults show rough eye phenotype with a relatively normal eye size and severe ommatidia loss (Fig 4C).

**Table 1:** Phenotypes associated with ectopic expression of *ey* and *RpPax6*.

	ey-GAL4	GMR-GAL4	dpp-GAL4	da-GAL4
Expression	Eye	Behind the morphogenetic furrow in the eye disc.	Limited portion of in all of the imaginal discs	Ubiquitously
UAS-ey	Pink eyes with non-uniformly reduced size(Fig 4G and 4H)		Pupal lethal (100%) Ectopic eyes on wings, Legs and head(Fig 4E).	
UAS-RpPax6	Pupal lethal (100%) Abnormal eye-antennal discs in third instar stage (Fig 5C). Headless phenotype in dead pharate (Fig4D).	Pupal lethality (92.1%) rough eye and severe ommatidia loss (Fig 4C).	Larval and pupal lethal (100%) Under development in the eyes, wings and legs.	Embryonic lethal (100%)

To delineate the function of *RpPax6* the *Drosophila Pax6* homologue gene *ey* was expressed using *dpp-GAL4* and *ey-GAL4*. The result show that expression of *ey* with *dpp-GAL4* leads to significant pupal lethality. Flies dissected from the pupal case show the formation of ectopic eyes on wings, legs and head. Expression of *ey* with *ey-GAL4* produced pink eyes with non-uniformly reduced size. As shown in Fig 4G and 4H, the left eye of the fly was significantly reduced in size whereas the right eye was slightly reduced.

### *RpPax6* expression induced apoptosis

**Fig 6:**
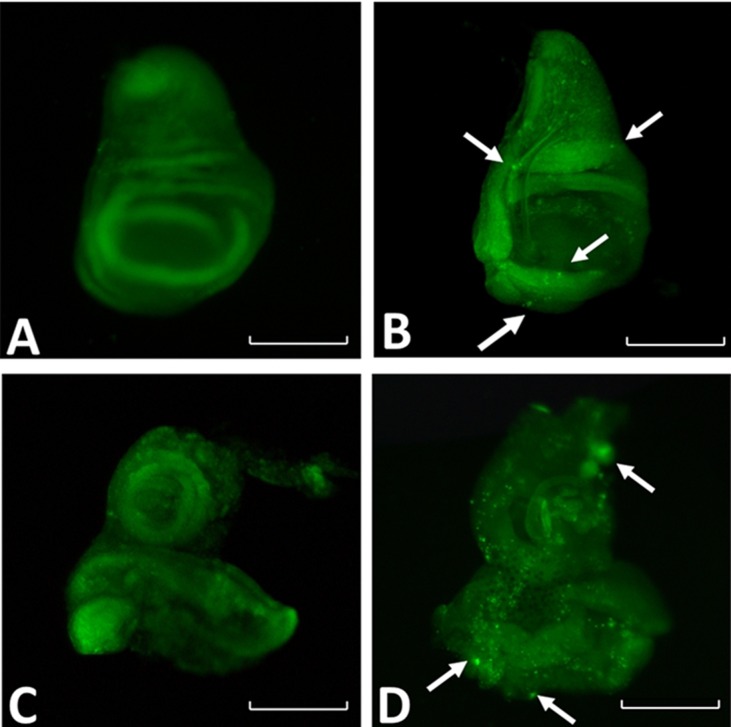
Expression of *RpPax6* promotes apoptotic cells were identified by acridine orange staining. Arrow points to a cluster of apoptotic cells. All discs are reproduced at the same magnification. Scale bars = 100 μm. (A) Third instar wing disc of *UAS-RpPax6/da-GAL4*,*tub-GAL80*^*ts*^ reared continuously at 18°C exhibit no cell death as indicated by a lack of nuclear acridine orange staining. (B) Expression of *RpPax6* for 24 h (*UAS-RpPax6/da-GAL4*,*tub-GAL80*^*ts*^, shifted to 29°C) lead to cell death in third instar wing disc as indicated by nuclear acridine orange staining (green spots). (C) Third instar of eye-antenna discs of *UAS-RpPax6/da-GAL4*,*tub-GAL80*^*ts*^ reared continuously at 18°C show a few cell death. (D) Expression of *RpPax6* for 24 h (*UAS-RpPax6/da-GAL4*,*tub-GAL80*^*ts*^, shifted to 29°C) lead to high frequency of apoptotic cells in third instar eye-antenna discs.

Expression of *RpPax6* using *ey-GAL4* cause abnormal eye-antennal disc, defects in the head region, and death during the pupal stage. The reduced size of the fly head could be due to cell death induced by *RpPax6*. This is supported by the observation that throughout expression of *RpPax6* with *da-GAL4* caused earlier and more severe lethality than restricted expression with *ey-GAL4*, *GMR-GAL4* and *dpp-GAL4* (Table 1). To investigate the effects of *RpPax6* expression to the cell death, *y w;+/+;UAS-RpPax6* stock was crossed to the line *y w;+/+;da-GAL4*,*tub-GAL80*^*ts*^. The cross was reared at 18°C for 120 h to ensure tight suppression of *RpPax6* expression until third instar stage. And then *RpPax6* was induced by 29°C temperature shift to inactivate GAL80 and subsequently activate Gal4 activity. The third instar larval wing and eye-antenna discs were examined by using acridine orange staining, a vital dye that preferentially labels apoptotic cells [41]. Larva with temperature shift-up had higher level of dying cells in both discs than those raised continuously reared at 18°C (Fig 6), suggesting *RpPax6* expression causes cell death.

### RpPax6 can not rescue ey^2^ mutant

**Fig 7:**
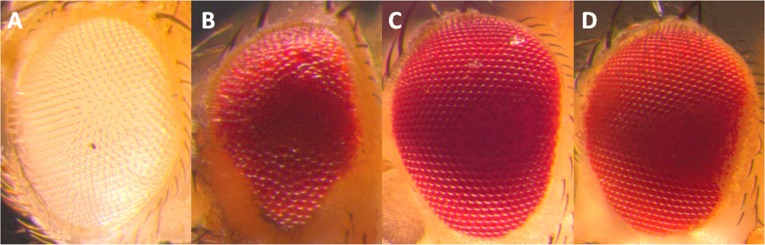
Mutant rescue by targeted expression of ey and mPax6. (A) eye of *y w* fly. The ey^2^ mutant phenotype (small eye, see Fig B) can be completely rescued by targeted expression of ey (C) and mPax6 (D) driven by ey-GAL4.

Rescue experiments were set up to assess the importance of the RpPax6 in vivo. To allow eye-specific expression we used ey-Gal4 to drive UAS-ey, UAS-mPax6 (mouse Pax6) and UAS- RpPax6 genes in ey^2^ mutant background. ey mutation ey^2^ is amorphic for ey function in the eye disc. Flies homozygous for ey^2^ have reduced eyes. Expression of RpPax6 induced by ey-GAL4 in an ey^2^ mutant background caused pupal lethality. The dead pharates exhibited headless phenotype and the third instar larvae showed abnormal morphology of eye-antennal discs (data not shown), as the condition in UAS-RpPax6/ey-GAL4 flies. In addition, in the control cross, in which both full-length ey and mPax6 proteins were misexpressed in the same genetic background, the ey^2^ mutant was fully rescued. The eyes were morphologically normal and often of normal size (Fig 7). The rescue results suggest that RpPax6 expression can not induce eye development like ey and mPax6 do.

### Inhibition of apoptosis partially rescued *RpPax6-*induced headless phenotype

**Fig 8:**
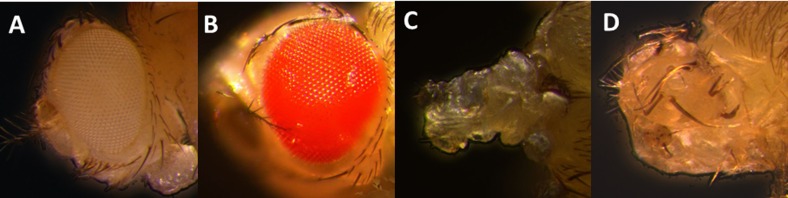
Eye phenotype caused by expression of RpPax6 is partially rescued by inhibition of apoptosis (A, B) head of *y w* and *UAS-RpPax6* pharate. (C) *UAS-RpPax6/ey-GAL4* pharate with most head structures and both eyes absent. (D) *UAS-RpPax6/ey-GAL4* pharate headless phenotype can be largely rescued by coexpression of p35, an inhibitor of apoptosis.

Our results revealed that flies expressing *RpPax6* with *ey-GAL4* die as pharates showing a headless phenotype(Fig 4D). If the defects are caused by apoptosis, it might be possible to rescue the headless phenotype by inhibition of apoptosis. To test this possibility the effect of a well-characterized anti-apoptotic viral protein P35 that inhibits downstream effector caspases was assessed. Coexpression of *RpPax6* together with *P35* with the use of *ey-GAL4* resulted in the suppression of the *RpPax6* induced defects. Although the rescued flies cannot eclose, almost all pharate adults exhibit an eyeless phenotype with most head structures recovered (Fig 8D). Reduction in the severity of the defect strongly supports a role for caspase-dependent apoptotic cell death in *RpPax6* induced abnormalities.

## Discussion

Covering nearly two-thirds of the Earth’s surface, deep-sea regions are known to harbor complex communities with impressively high numbers of species, showing remarkable morphological and physiological adaptations [46–48]. Due to the difficulties in the sampling and preserving of individual specimens, molecular studies of deep-sea organisms are still rare. *R*. *piscesae* is the foundation species in many Juan de Fuca Ridge hydrothermal vent communities [36, 37] within depths ranging from 1570–3250 m [35, 49–51]. *R*. *piscesaeis* presumed to have lost eyes during evolution because eyes are widespread throughout many annelid taxa [23, 52–54]. Likewise absence of eyes is observed in many species living in ocean depths beyond the penetration of daylight. These blind species might have dispensed with vision due to the lack of selective advantage in a dark environment [55, 56].

*Pax6* play a central role in the core gene regulatory network that governs the development of the eye, a function that is well conserved in both invertebrates and vertebrates. The eye regulatory genes including *ey*, *toy*, *so*, *eya* and *dac* control early eye development in *Drosophila* [57]. *Toy* acts upstream to induce *ey* expression [58, 59]. *Ey* activates expression of *so*, *eya* and *dac* [13, 43, 60]. The latter three are part of a positive feedback loop controlling *ey* expression [61–63] and further regulate more peripheral genes in the eye development network such as atonal (*Ato*) [56, 64]. The gene networks have meanwhile also been extended to the respective mammalian proteins [65–67]. Thus Pax6 proteins (ey and toy) function upstream in eye gene hierarchies, orchestrating all downstream events. To test if *Pax6* is also involved in eye absence in deep sea organisms, we identified and characterized *Pax6* of *R*. *piscesae*. Comparison of the deduced amino acid sequence with Pax6 proteins from other species revealed that two characteristic domains (PD and HD) are highly conserved, indicating selection pressure was exerted due to functional constraints.

*Drosophila Pax6* homolog *ey* has been shown to bind directly to the *so* promoter and activate *so* expression [13]. However, our functional analysis by dual luciferase assay revealed that *RpPax6* can directly target on *so*, not as activator but as repressor. As DNA binding transcription factor, *Pax* genes regulate the development of multiple organs through the utilization of PD and HD combination for target gene promoter recognition [68]. The results (Fig 3) non-conserved C region moiety plays crucial role in the suppression of *so* promoter activity. Previous study has revealed that transactivation domain in ey as well as Pax6 of quail are both located in the c-terminus of the protein [69, 70]. It is possible that the C-terminal moiety of *RpPax6* achieved repression ability either directly or by activation of a repressor. In addition ey has been found to be a putative transcriptional repressor. The repressive activity lies in the linker region between paired domain and homeodomain [69]. Our result show RpPax6 with N-terminal moiety swapped with the corresponding portion of ey (eyN+RpPax6C and eyPD+RpPax6HD) had little impact on the activity of the *so* promoter (Fig 3), indicating the presence of repressive activity within the N-terminal moiety of *RpPax6*.

The ability to induce ectopic eyes through *Pax6* misexpression has been demonstrated in *Drosophila* and vertebrates [12]. *Pax6* genes of various species including *C*. *elegans* [71] mouse, *Drosophila* [72], sea squirt [11], squid [10], and lancelet [71] are capable to induce supernumerary eyes upon targeted ectopic expression by means of the GAL4-system in *D*. *Melanogaster* [8, 10]. While the reciprocal experiment, expression of *Drosophila ey* and its paralog twin of eyeless *(toy*) genes in *Xenopus* embryos, induces the development of vertebrate eye structures [73]. Our experiments show flies expression of *UAS-ey* under the control of *dpp-GAL4* generated many ectopic eye structures on wings, legs and head, in line with previous research [8]. By contrast, none of the *UAS-RpPax6* transgenic lines was able to induce ectopic eye morphogenesis under the same conditions. It is conceivable that *RpPax6* expression cause repression of *so* and lead to the failure to trigger the eye formation process.

To elucidate the role of RpPax6 in eye developmental cascade, we misexpressed ey, mPax6 and RpPax6 in an ey mutant background. Rescuing the ey^2^ mutant by ey and mPax6 leads to full recovery of eye size. However, expression of RpPax6 in the same background is lethal with a lethal phase during the pupal stage, in agreement with the lethal phase of the severe ey mutant eyD. eyD mutants has been described to develop into fully formed headless adults that fail to eclose from their pupal cases (pharate adults) [59]. Interestingly, similar headless phenotypes was also produced in pharates of RpPax6 expressed ey^2^ mutant. The EyD protein not only lacks the entire homeodomain, but also 660 amino acids in the C-terminus [74] show that overexpression of ey can rescue the lethality of homozygous eyD mutants and also suppress the eyD phenotype and partially restore head development. It has been suggested that the remaining paired domain is be able to bind to the cluster of binding sites within the eye enhancer of so, but the modifications in the C-terminus might interfere with normal transcriptional activation. Similarly, our Luciferase reporter assay revealed that RpPax6 protein suppresses the transcription of sine oculis (so) and the C-terminal of RpPax6 plays crucial role in the repressive activity. We postulate that RpPax6 expression induced by ey-GAL4 might interfere with the developmental pathway during eye-antenna disc formation and lead to the headless phenotype.

In *D*. *melanogaster*, the compound eyes and most head structures (head capsule, antenna, ocelli) develop entirely from the eye-antennal discs [75], in which process *ey* gene is involved in cell proliferation, differentiation and migration/adhesion [76, 77]. The headless phenotype characterized by the lack of structures derived from eye-antennal discs suggests massive cell death during development [56, 78]. As evident from acridine orange staining of *RpPax6* expressed larva and rescue of headless by the expression of the baculovirus P35 protein in eye-antennal discs (Fig 8D), the headless phenotype of *UAS-RpPax6/ey-GAL4* pharates results from the induction of apoptosis by expression of *RpPax6*. Paradoxically, authentic *Pax* genes are associated with differentiation, proliferation and anti-apoptosis during development process [76, 77]. Inhibition of *ey* [58, 79] as well as other *Pax* genes including *Pax2*, *Pax8* [80], *Pax3* and *Pax7* [81] induce apoptosis. Interestingly, *Pax6* is involved in the eye regression of a blind cave form of the teleost *Astyanax mexicanus*. *Pax6* sequence is found to be identical in the eyeless cave form (cavefish) and eyed surface form (surface fish) [82]. However, during embryonic development in cavefish increased expression of *sonic hedgehog* (*Shh*) suppresses *Pax6* and increases expression of *Shh-regulated* genes, which further results in lens apoptosis and eye degeneration. The apoptosis and *Pax6* down regulation are common to several independently derived cavefish populations, suggesting the importance in the evolution of eye degeneration [83–86]. The results of our study indicate that alternation of *Pax6* function may cause the activation of apoptosis and further contribute the blindness in vestimentiferan tubeworms. Fly models provide powerful genetic systems to dissect the role of transcription factor change in developmental evolution. In the present study we have used this tool to provide some initial molecular insights into the mechanisms of transcriptional regulation of RpPax6. Further in vivo investigations are necessary to validate the function in the normal context.
